# Strategic research leadership and research capacity enhancement in Ugandan universities: a systematic review

**DOI:** 10.12688/f1000research.183310.1

**Published:** 2026-06-09

**Authors:** Jerald Kule, Prashant Bhimsen Pagare

**Affiliations:** 1 Education, Ibanda University, Ibanda, Uganda, 35, Uganda; 2 Education, Dr Babasaheb Ambedkar Marathwada University, Aurangabad, Maharashtra, 431004, India

**Keywords:** Strategic research leadership, research capacity enhancement, Ugandan universities, research equity and inclusion, research capacity framework, research capacity model

## Abstract

**Background:**

Research capacity in Ugandan universities remains underdeveloped, reflecting a wider African challenge where the continent contributes barely 1% of global research output. Despite this, limited studies evaluate existing research capacity enhancement frameworks and models that focus on Ugandan universities. Therefore, this systematic review examined the scope of such frameworks, their conceptualisation of strategic research leadership (SRL), the mechanisms through which they link leadership practices to measurable research outcomes, and the limitations in their application.

**Methodology:**

A systematic review was conducted using PRISMA 2020 guidelines. Studies were identified through Google Scholar, Semantic Scholar, and ProQuest. Of 432 records screened, only nine met the inclusion criteria. Risk of bias was minimised through independent dual review, consensus, and arbitration by a third reviewer. Thematic analysis was used to synthesise findings.

**Results and Discussion:**

The review highlights a scarcity of context-specific frameworks, with most focusing on public universities, faculty-led research, and health sciences, while neglecting private universities, students, and humanities’ disciplines such as education. Integration of SRL constructs was limited, particularly in vision, policy, and culture. Leadership practices such as mentorship and collaboration enhanced measurable research outcomes, yet models faced sustainability, resource, and monitoring gaps. These findings underscore the need for comprehensive, inclusive frameworks that fully integrate SRL and support equity across institutions and disciplines. The review also lays the foundation for empirically developing and validating a context-specific SRL model to strengthen educational research capacity among students in Ugandan private universities, as these remain the most neglected categories in existing models and frameworks.

**Registration:**

This review was not registered in PROSPERO, as the database only accepts reviews addressing direct human health-related outcomes.

## Introduction

 Inadequate research capacity remains a profound developmental challenge that constrains Africa’s scientific advancement and economic progress. Unsurprisingly, the continent contributes barely 1% of global scientific output, despite accounting for 15% of the world’s population, 25% of the global disease burden, 60% of the extreme poor, and significant educational exclusions ( Dabou et al., 2026; Olufadewa et al., 2020; Sinha et al., 2024; Tamaki et al., 2026). Sub-Saharan Africa (SSA) faces similar limitations; for instance, Tamaki et al. (2026) reported that SSA accounted for only 2.7% of global citations in 2021. SSA, including Uganda, consequently experiences some of the most severe barriers to sustainable development—barriers that impactful research should be addressing ( Nakijoba & Awobamise, 2022; Zhang, 2024).

Globally, universities are increasingly expected to contribute to sustainable development through research, innovation, and teaching. In doing so, they shape policies and development agendas that drive societal progress. This impact, however, requires the active participation of all university systems. In developing regions such as Africa and SSA, universities’ contributions remain limited due to critically underdeveloped research capacity. In Uganda, the challenge is particularly acute: most universities operate under resource constraints and lack institutional and leadership support ( Hiire et al., 2020; Nakijoba & Awobamise, 2022; Obuku et al., 2018; Obuku et al., 2021). The situation is especially concerning in private universities, which constitute over 80% of Uganda’s universities ( National Council for Higher Education [NCHE], 2022). While these universities could significantly contribute to national development if empowered to engage in impactful research and innovation, they are often neglected. Public universities benefit disproportionately from government funding and policy attention, leaving private institutions to struggle with leadership, capacity, and resource challenges ( Obuku et al., 2021). This imbalance reflects structural, exclusionary, and leadership gaps that weaken Ugandan universities’ contributions to knowledge creation, research, teaching, and sustainable development.

Previous studies have attempted to address the challenge of low research capacity in Ugandan universities ( Hiire et al., 2020; Khisa et al., 2019; Manabe et al., 2019; Nanvuma et al., 2025; Obuku et al., 2018; Obuku et al., 2021; Zachariah et al., 2016, 2022). However, these efforts reveal limited localised, cohesive, and context-specific frameworks for enhancing research capacity. A disconnect persists between broad global capacity-building models and the highly specific, resource-constrained realities faced by universities in low- and middle-income countries (LMICs) such as Uganda ( Busse et al., 2022; Véliz et al., 2023). Consequently, many universities lack empirically grounded models to guide their research development. Importantly, there is limited examination of existing frameworks within the Ugandan context. Addressing these gaps is essential to empower universities to contribute meaningfully to research, teaching, innovation, and national development.

This study systematically reviews existing models and frameworks for research enhancement in Ugandan universities. Specifically, it examines: (1) the scope of research capacity enhancement models and frameworks; (2) how strategic research leadership (SRL) is conceptualised within these models; (3) the mechanisms through which leadership practice is linked to measurable research capacity outcomes; and (4) the gaps or limitations that emerge in their application within Ugandan universities?

SRL is central to this review, as it is proposed as a solution to inadequate research capacity. SRL represents an innovative integration of strategic leadership and research leadership. Strategic leadership refers to the deliberate practice of guiding organisations towards long-term success through vision development, decision-making, inspiration, and resource alignment to ensure stability, adaptability, and future achievement ( Davies & Davis, 2004; Samimi et al., 2022). Research leadership, by contrast, involves shaping and supporting research activities to achieve scholarly goals and impact, often through mentorship, collaboration, resource mobilisation, and quality enhancement ( Flinders, 2022). The integration of these dimensions produces SRL—a unique, under-researched leadership approach that deliberately shapes and supports research activities through vision-setting, policy development, mentorship, resource mobilisation, collaboration, and culture-building. SRL thus strengthens research capacity and enhances quality, productivity, and impact.

By advancing this perspective, the study contributes to intellectual discourse on research leadership and capacity enhancement in Ugandan universities. It identifies SRL as a crucial leadership dimension capable of addressing persistent capacity gaps. The findings of this systematic review, particularly the identified limitations in empirical, context-specific models, also lay the foundation for empirically developing and validating a context-specific SRL model to strengthen educational research capacity among students in Ugandan private universities. More broadly, the study holds relevance for universities seeking to reform research cultures through leadership innovation in Uganda, SSA, and other developing regions where research productivity and impact remain constrained.

Finally, the paper follows the IMRaD and PRISMA 2020 structure, presenting the introduction, methodology, results, and discussion.

## Methodology

### Research design

We conducted a systematic review in accordance with the Preferred Reporting Items for Systematic Reviews and Meta-Analyses (PRISMA) 2020 guidelines. The review examined the scope of research capacity enhancement frameworks and models, their conceptualisation of strategic research leadership (SRL), the mechanisms linking leadership practice to measurable research capacity outcomes, and the limitations of these models in the context of Ugandan universities.

### Search methods

Peer-reviewed studies containing relevant models and frameworks were identified through Google Scholar, Semantic Scholar, and ProQuest. Employing multiple databases enriched the search process by improving recall, broadening coverage, and maximising available data. ProQuest provided a user-friendly interface, in-depth search capabilities, and efficient document sorting by publication date and relevance, while also facilitating the exclusion of duplicates. Semantic Scholar functioned as a specialised tool for mapping, filtering, and identifying relevant literature, ensuring that key scholarly contributions were not overlooked. Google Scholar enabled the identification of highly cited literature, offered citation metrics, and facilitated the saving of relevant articles for further analysis.

### Search strategy

The following Boolean search terms were applied: (“research capacity building model” OR “research capacity building framework” OR “research capacity enhancement model” OR “research capacity enhancement framework” OR “research capacity development model” OR “research capacity development framework” OR “research capacity strengthening model” OR “research capacity strengthening framework”) AND (“higher education” OR “universities” OR “university”) AND (“Uganda” OR “Africa”). This strategy was applied across the three databases. Initially, 432 studies were identified; however, only 9 met the inclusion criteria and were retained for review.

### Selection of studies

Study selection followed the inclusion and exclusion criteria strictly. The PRISMA 2020 flow process guided the stages of identification, screening, eligibility, and inclusion. Titles and abstracts were scanned, followed by full-text reviews of potentially eligible studies. Results were collated in Mendeley, which facilitated duplicate removal. Disagreements during study assessment were resolved through consensus.

### Inclusion and exclusion criteria

Studies were excluded if they addressed unrelated themes, lacked models or frameworks, or were not relevant to research capacity enhancement in Ugandan universities. Additionally, studies published prior to 2015 were excluded. The inclusion and exclusion criteria are summarised in  Table 1.

**Table 1.** Inclusion and exclusion criteria.

Parameter	Included	Excluded
Theme	Themes related to research capacity enhancement	Themes unrelated to research capacity enhancement
Model or framework	Having a research capacity enhancement model or framework.	No research capacity enhancement model or framework.
Context	Focusing on Ugandan universities	Unrelated to Ugandan universities
Study type	Peer reviewed. Yes	Not peer reviewed
Grey literature	No	Yes
Studies in predatory journals	No	Yes
Language	English	Other languages
Date of publication	January 1, 2015 – April 8, 2026	Prior to January 1, 2015
Accessibility	Open access	Subscription

### Quality assessment, risk of bias assessment, and data extraction

Data were independently extracted and analysed using a structured data extraction form. Any discrepancies encountered during the process were harmonised through discussion and consensus, and to further minimise risk of bias, a third independent reviewer was consulted to compare, assess, and resolve disagreements. The extraction form captured key elements including author and year, model or framework, strategic research leadership dimensions, mechanisms linking leadership practice to measurable research outcomes, limitations or gaps within the models, and scope (target university type, geographical context, target group, and target discipline).

## Results

The initial search for research capacity enhancement models or frameworks in Ugandan universities yielded 432 records across Google Scholar, Semantic Scholar, and ProQuest (see Figure 1). Following duplicate removal and screening for relevance and eligibility, only nine studies met the inclusion criteria and were retained for analysis. These studies were subjected to thematic synthesis, guided by four key questions: (1) what is the scope of research capacity enhancement models or frameworks in Ugandan universities? (2) how is strategic research leadership conceptualised within these models or frameworks? (3) through what mechanisms do these models link leadership practice to measurable research capacity outcomes? and (4) what gaps or limitations emerge in their application within the Ugandan context. The results of this synthesis are presented below.

**Figure 1.**
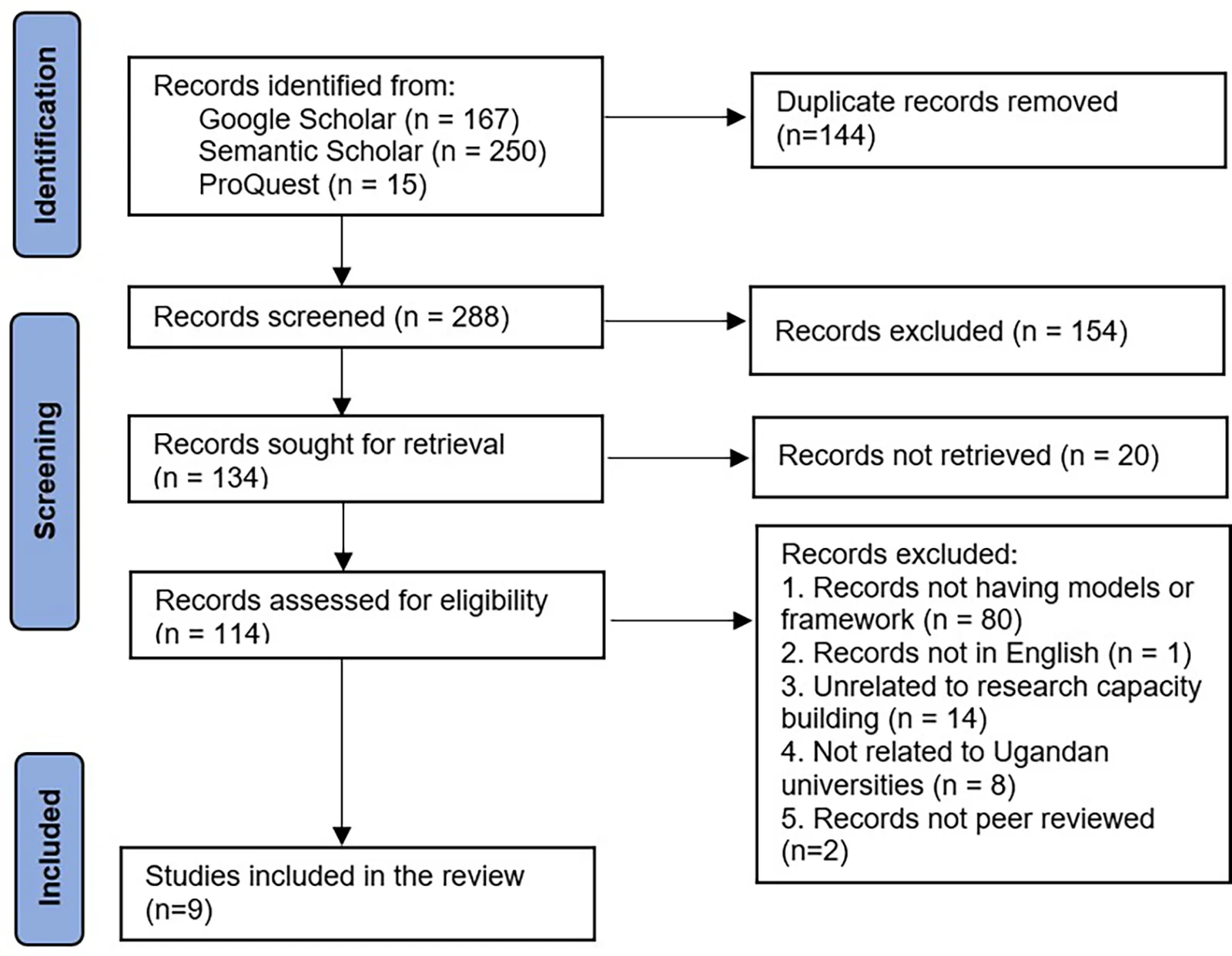
PRISMA 2020 flow diagram. *Note.* The diagram ( Figure 1) illustrates the total records identified from Google Scholar, Semantic Scholar, and ProQuest, and the subsequent screening, exclusion, and inclusion processes. In total, 432 records were initially identified; however, following duplicate removal and eligibility screening, only nine studies met the inclusion criteria and were retained for analysis.

### Question 1: What is the scope of research capacity enhancement models or frameworks in Ugandan universities?

In response to the first research question, we analysed the scope of research capacity enhancement models and frameworks in Ugandan universities. The analysis focused on their coverage of thematic areas in relation to university category (public or private), target groups (students or faculty), geographical region (national or continental), and disciplinary orientation. This approach enabled us to determine whether the models were broadly applicable across institutions or tailored to specific contexts, and whether they addressed diverse academic disciplines or concentrated on particular fields. The results of this analysis are summarised in  Table 2.

**Table 2.** Scope of research capacity enhancement models or frameworks.

Record	Framework/Model	University	Group	Region	Discipline
Manabe et al. 2019	Group Mentorship Model	Makerere University & other participating universities	Students	SSA & Uganda	Health
Muhumuza et al. 2022	Busitema University Research Model	Busitema University	General	Uganda	General
Ngongalah et al. 2019	CORE Africa framework (Collaboration for Research Excellence in Africa)	General	General	Africa	Health
Nanvuma et al. 2025	Capacity Building Model to nurture junior independent clinical researchers	Makerere University	Students	Uganda	Health
Perier et al. 2022	Workshop-based learning and networking approach	Makerere University	Students and junior faculty	Uganda	Health
Bebell et al. 2022	Train the trainer model	Public	General Health workers	SSA Uganda	Health
Zachariah et al. 2022	SORT IT model	General	Health workers/general	LMICs	Health
Mbwayo et al. 2022	Smart Africa capacity building framework	General	General	SSA (Ghana, Kenya, Uganda,	Health
Odeny et al. 2026	TIMER 2C	General	Health workers/General	MENA/SSA/Uganda	Health

The analysis of research capacity enhancement models and frameworks revealed notable variations in scope across university category, target group, discipline, and region. Most models were directed towards public universities, particularly Makerere University, with three frameworks (33.3%) focusing exclusively on it. One model (11.1%) targeted Busitema University, while another (11.1%) addressed public universities more broadly. In contrast, private universities received limited attention, except within generic models that encompassed universities and other professionals, especially in health-related fields. Overall, three general models accounted for 44.4% of the total. Regarding target groups, three models (33.3%) were general, three (33.3%) leaned towards health workers, one (11.1%) focused on both students and junior faculty, and two (22.2%) concentrated solely on students. Disciplinary scope was heavily skewed towards health research, with eight models (88.8%) addressing this domain, while only one (11.1%) adopted a general orientation without disciplinary restriction. Regional coverage also varied: three models (33%) focused specifically on Uganda, whereas six (67%) adopted a broader perspective, situating Uganda within the wider contexts of low- and middle-income countries, Sub-Saharan Africa, or Africa as a whole.

### Question 2: How is strategic research leadership conceptualised within research capacity enhancement models or frameworks in Ugandan universities?

SRL practices emerged as key thematic areas that illustrate how these models or frameworks conceptualise strategic research leadership. The thematic areas include vision, collaboration and networking, mentorship, resource mobilisation and allocation, policy, and culture.  Table 3 summarises the specific SRL concepts or constructs emphasised by each model or framework.

**Table 3.** Conceptualisation of SRL across models or frameworks.

Framework/Model	Research vision	Research mentorship	Research collaboration	Resources allocation	Research policy	Research culture
Group Mentorship Model						
Busitema University Research Model						
CORE Africa framework						
Capacity Building Model to nurture junior independent clinical researchers						
Workshop-based learning and networking approach						
Train the trainer model						
SORT IT model						
Smart Africa capacity building framework						
TIMER-2C						

*Note*: The shaded areas in  Table 3 indicate the specific SRL practices or concepts emphasised by each of the models or frameworks.

### Research vision

This emerged as one of the SRL concepts emphasised in models or frameworks for research capacity enhancement in Ugandan universities. Within this thematic area, universities deliberately establish a clear and shared direction for strengthening research capacity. Research vision was explicitly highlighted by one model (11.1%)—the Busitema University Research Model (BURM)—which conceptualises it as the responsibility of university leaders to develop a defined research agenda and strategy aimed at enhancing research output and publication. Such a vision sets research goals, aligns them with institutional priorities, and communicates them in ways that inspire engagement from all stakeholders. Although some other models or frameworks demonstrate elements of sustainability in research efforts, they do not explicitly identify research vision development as a core pillar for enhancing research capacity in Ugandan universities.

### Research mentorship

Mentorship emerged as one of the most prominent SRL practices, with all reviewed models and frameworks identifying it as a key construct for enhancing research capacity. These frameworks conceptualise mentorship as a multidimensional mechanism that extends beyond the traditional supervisor–student relationship to encompass interaction, collaboration, peer mentorship, and sustainability. Collectively, they redefine mentorship through three distinct dimensions.

The first group of models, such as the SORT IT model and the Busitema University Research Model (BURM), conceptualises mentorship as a structured and performance-driven pipeline. Here, mentorship functions as a formal tool for institutional accountability, succession planning, and the achievement of specific research output metrics, including publications and policy briefs ( Zachariah et al., 2022; Muhumuza et al., 2022). In contrast, models such as the Group Mentorship Model, Capacity Building Model, SMART Africa, and the Workshop-Based Model view mentorship as a collaborative and horizontal system that prioritises peer-to-peer and multi-tiered interactions. These frameworks emphasise bi-directional knowledge exchange and highlight international collaborations as central to large-scale capacity building ( Manabe et al., 2019; Mbwayo et al., 2022; Nanvuma et al., 2025; Perier et al., 2022). Finally, models including the Capacity Building Model, CORE Africa framework, Train-the-Trainer model, and TIMER-2C conceptualise mentorship as a holistic and ecological endeavour. In this perspective, mentors act as sponsors and counsellors who safeguard researchers’ time and psychological resilience against institutional instability ( Bebell et al., 2022; Nanvuma et al., 2025; Ngongalah et al., 2019; Odeny et al., 2026).

Overall, mentorship is framed as guidance, counselling, training, leadership, supervision, interaction, and empowerment—enabling researchers to, in turn, empower others. These models demonstrate a shift from rigid, top-down hierarchies towards more flexible approaches centred on interaction, peer mentorship, and sustainability. Modern mentorship is increasingly human-centred, focusing not only on research outputs but also on nurturing the researcher. It fosters independence, stewardship, self-reliance, and the intentional creation of a supportive research community. In doing so, mentorship builds responsibility and empowerment, ensuring that mentees are prepared to succeed and eventually replace their mentors. Thus, the models suggest that strategic research leadership is not merely the management of tasks but the cultivation of a resilient, intergenerational community of researchers.

### Research collaboration

Collaboration also emerged as a key construct of SRL, with all models explicitly identifying it as a crucial dimension for enhancing research capacity. Conceptually, collaboration is presented as a multi-stakeholder, partnership-driven approach encompassing mentorship, knowledge translation, skill acquisition, and resource management ( Zachariah et al., 2022; Bebell et al., 2022; Manabe et al., 2019; Mbwayo et al., 2022; Muhumuza et al., 2022; Nanvuma et al., 2025; Ngongalah et al., 2019; Odeny et al., 2026; Perier et al., 2022). Accordingly, the models and frameworks generally portray collaboration as a vehicle through which research leaders can address gaps in research resources ( Manabe et al., 2019), skill acquisition ( Ngongalah et al., 2019; Perier et al., 2022), and mentorship ( Bebell et al., 2022) by networking, partnering, cooperating, or working together with local, national, and international partners. Collaboration may also take the form of peer-to-peer ( Manabe et al., 2019; Nanvuma et al., 2025) or top-down structures ( Nanvuma et al., 2025), and is recognised as a means of enhancing research output ( Muhumuza et al., 2022). Additionally, while the workshop-based model emphasises didactic transfer, peer-driven frameworks such as CORE Africa and structural models such as SMART-Africa prioritise long-term, relational, team-based, and policy-engaged research enhancement. However, although collaboration is widely acknowledged as effective in grant writing, navigating power imbalances remains necessary to successfully align research enhancement with local revenue. Therefore, strategic research leaders should actively institutionalise collaboration as a cornerstone of research capacity enhancement to ensure that partnerships translate into sustainable skills, empowerment, resources, and outputs.

### Research resource allocation

Resources constitute another important construct of SRL, as they are explicitly incorporated in all the models and frameworks reviewed (Workshop-Based Model, SORT IT Model, Capacity Building Model, Train-the-Trainer Model, CORE Africa Framework, BU Research Model, Group Mentorship Model, TIMER-2C Framework, SMART-Africa). This construct is generally conceptualised as the means and facilities that support the research process, including financial resources such as funding, physical resources such as laboratories and libraries, human resources such as supervisors and mentors, and digital resources such as internet connectivity—all of which facilitate a sustainable research culture ( Zachariah et al., 2022; Bebell et al., 2022; Manabe et al., 2019; Mbwayo et al., 2022; Muhumuza et al., 2022; Nanvuma et al., 2025; Ngongalah et al., 2019; Odeny et al., 2026; Perier et al., 2022). For instance, according to Muhumuza et al. (2022), the BU Research Model considers resources as inputs that enable research leaders to motivate researchers and sustain outputs such as articles, book chapters, and books. Similarly, under the Capacity Building Model and the Group Mentorship Model, fellowships and mentors are provided to nurture junior independent clinical researchers and facilitate research projects ( Manabe et al., 2019; Nanvuma et al., 2025). The SMART-Africa framework also addresses research capacity enhancement in mental health systems and implementation research by establishing a regional transdisciplinary collaborative centre, which serves as a critical resource in this endeavour ( Mbwayo et al., 2022). However, although these models depict research resources as multidimensional and crucial for sustaining research capacity enhancement efforts, they also highlight persistent constraints, particularly within Ugandan universities. The role of SRL is therefore to mobilise these resources and allocate them efficiently to ensure the sustainability of research activities.

### Research policy

Research policy was conceptualised by two models—the Busitema University Research Model ( Muhumuza et al., 2022) and the SMART Africa Capacity Building Framework ( Mbwayo et al., 2022). Within the SMART Africa framework, research policy is presented as an integral component of leadership, used by leaders to guide and influence capacity-building interventions in research and health. In this model, policy is framed as a collaborative and context-driven process aimed at strengthening child mental health systems in Sub-Saharan Africa, including Uganda. It bridges the gap between evidence-based interventions and policy, thereby strengthening local research capacity, fostering transdisciplinary partnerships, and integrating mental health research into existing policies for institutional support. The Busitema University Research Model views research policy as guidelines, laws, and frameworks that support the enhancement of research quality and output. It is presented as one of the strong pillars of research enhancement processes, providing both mandate and guidance for other interventions. Policy, in this context, supports leaders and researchers by offering direction on what to do and how to proceed. The model emphasises research policy as a driver for strengthening academic staff capacity, promoting mentorship, and managing resources to facilitate research productivity. Taken together, these two frameworks suggest that evidence-based research policy should serve as a guiding framework that prioritises, supports, and directs research capacity enhancement interventions.

### Research culture

The TIMER-2C framework ( Odeny et al., 2026) is the only model that explicitly mentions research culture, emphasising the enhancement of research capacity by focusing efforts on developing a strong institutional research culture. This framework specifically addresses barriers to research culture and productivity among local healthcare providers in the Middle East and Sub-Saharan Africa, making culture one of its central pillars. It conceptualises culture as a comprehensive, multilevel leadership construct that dictates the success of all other capacity-building interventions. According to the model, a positive research culture fosters sustainable research capacity by integrating collaboration, systemic recognition, the value of research, institutional support, transformation, motivation, stabilisation, and protection of research interests against institutional and political instability. Research culture situates research and researchers within a supportive and conducive environment, enabling them to thrive sustainably. Thus, a robust research culture is presented as clear evidence of supportive SRL.

The models and frameworks reviewed show that strategic research leadership (SRL) is understood through practices such as vision, collaboration, mentorship, resource mobilisation, policy, and culture. However, the conceptualisation within individual models is often shallow, focusing narrowly on one or two dimensions. It is only when all the models are considered together that a richer and more comprehensive picture of SRL emerges—one that highlights leadership as both guiding research processes and building sustainable, resilient academic communities in Ugandan universities.

### Question 3: Through what mechanisms do research capacity enhancement models or frameworks in Ugandan universities link leadership practice to measurable research capacity outcomes?

The reviewed research capacity enhancement frameworks and models in Ugandan universities link leadership practices such as mentorship, resource mobilisation and allocation, policy development, and vision setting to measurable research outcomes—including publications, skills, grants, and policy influence—through structured, participatory, and context-tailored mechanisms. These frameworks and models move beyond training to institutionalise research, promote transformational leadership, strengthen supportive research environments, and implement systematic evaluation metrics. Thematic areas that emerged under this question include structured mentorship, strategic evaluation and quality management, resource management, regional and international collaborations, and action-oriented research training, as presented below.

### Structured mentorship

All frameworks and models (Group Mentorship Model, Busitema University Research Model, Capacity Building Model, Train-the-Trainer, SMART-Africa Capacity Building Framework, Workshop-Based Model, SORT IT Model, TIMER-2C) propose that leaders employ top-down, peer-to-peer, or combined research support mechanisms to enhance capacity and achieve measurable outcomes such as increased grant success, more publications, conference presentations, improved research skills, and greater retention of junior researchers. This mechanism blends formal hierarchy with peer-to-peer mentoring to ensure knowledge transfer, reduce research isolation, and foster succession planning. Leaders should participate in or facilitate these processes so that senior staff and students, especially PhD candidates, can provide mentorship. For instance, the Group Mentorship Model ( Manabe et al., 2019) and the Capacity Building Model ( Nanvuma et al., 2025) combine formal hierarchy with peer-to-peer mechanisms to ensure effective mentoring. Through this mechanism, the Group Mentorship Model produced 169 peer-reviewed publications, co-supervised 65 master’s students by PhD students, and trained 17 PhD students ( Manabe et al., 2019). Similarly, Muhumuza et al. (2022) show that under the Busitema University Research Model, senior academic staff mentoring junior staff and postgraduate students (hierarchical top-down mentorship) resulted in measurable outputs such as books, articles, and book chapters, thereby improving the university’s ranking.

### Action-oriented research training

Models and frameworks, particularly SORT IT, the Capacity Building Model, Workshop-Based Model, Train-the-Trainer, and Group Mentorship Model, emphasise hands-on training in research skills such as writing, presentation, data analysis, and publishing. Through this mechanism, universities achieve high completion rates of publishable manuscripts, policy influence, and peer-reviewed journal articles. Sustainability is realised when graduates of these programmes train and mentor others. Under the SORT IT model ( Zachariah et al., 2022, 2016), leadership shifts from theoretical workshops to operational research where participants address practical, institution-specific problems. Training is thus embedded into daily tasks and aligned with national research priorities. Bebell et al. (2022) propose that under the Train-the-Trainer model, leaders should emphasise targeted training so that trainees can train others, ensuring sustainability of perinatal pathology research capacity in Sub-Saharan Africa. The Group Mentorship Model ( Manabe et al., 2019) focuses on training postgraduate students to sustain research capacity enhancement efforts. These trainings strengthen students’ research capacity, enabling them to complete projects, publish, and engage in peer-to-peer mentorship. In this way, leadership support for training directly translates into tangible outcomes that strengthen the research capacity and impact of universities.

### Resource management

Research leaders focus on creating supportive infrastructure such as dedicated research offices, e-supervision platforms, and standardised management policies to improve measurable outputs, including publications and timely completion of postgraduate studies. Resource management also encompasses human resources such as supervisors, physical resources like laboratories and libraries, and financial resources such as grants. For example, senior supervisors and researchers help junior colleagues and students complete projects and produce publishable manuscripts, while funding enables mentored researchers to cover article processing charges for quality journals ( Manabe et al., 2019; Muhumuza et al., 2022). This mechanism is illustrated in the Busitema University Research Model and the Group Mentorship Model, among others. Leaders are responsible for mobilising and allocating resources to such activities and ensuring accountability, thereby sustaining measurable research outcomes.

### Regional and international collaboration

Collaboration is an essential vehicle for delivering research outcomes. Research leaders facilitate partnerships, networks, and collaborations that provide universities with access to advanced technology, expertise, funding, and training, ensuring sustainability of research capacity development. Makerere University has benefitted from such partnerships through the Group Mentorship Model under the Medical Education Partnership Initiative–Medical Education for Equitable Services to All Ugandans (MEPI-MESAU) programme. Managed by the leadership of Makerere University College of Health Sciences and funded by international partners (U.S., PEPFAR, NIH), the programme enables Makerere to collaborate with other regional universities—including Kampala International University, Busitema University, Gulu University, and Mbarara University of Science and Technology—to enhance medical education and research capacity. Through the Group Mentorship Model, the programme has produced PhD graduates, publications, conferences, and policy interventions. Other models where international and regional collaborations facilitate training, skills development, resources, publications, and conferences include the Train-the-Trainer Model ( Bebell et al., 2022), SMART-Africa Capacity Building Model ( Mbwayo et al., 2022), TIMER-2C ( Odeny et al., 2026), and Workshop-Based Learning Model. Leaders can therefore leverage collaborations to expand funding streams, develop cross-institutional initiatives, and increase international collaborative publications.

### Strategic evaluation and quality management

Research leadership implements regular monitoring mechanisms such as pre-training and post-training tests, research ethics committees, quality assurance committees, appraisals, and accountability processes to ensure that institutional, faculty, and student-led research is produced, evaluated, regulated, and supported. These mechanisms enable universities to build capacity for producing quality research, publications, proposals, grants, and presentations. Strategic evaluation and quality management reduce the time required to complete projects while maintaining standards. For example, faster ethical approval processes can motivate researchers to complete projects on time. Such mechanisms also ensure compliance with international standards and funder agreements. Under the TIMER-2C model ( Odeny et al., 2026), recognition is emphasised as an important aspect of research capacity enhancement. Achievements are evaluated at personal, interpersonal, and institutional levels to identify successes and reward them appropriately.

### Question 4: What gaps or limitations emerge in the application of these models in the context of Ugandan universities?

The review of research capacity enhancement models and frameworks in Ugandan universities produced nine thematic areas of gaps and limitations. These include limited inclusion and scope, limited sustainability, resource constraints, limited integration of SRL constructs, limited research application mechanisms, individual versus institutional capacity gaps, and monitoring and evaluation gaps. Some of these limitations are institutional, while others lie in the design of the models and frameworks themselves.

### Limited inclusion and scope

Most models do not approach research capacity enhancement as a holistic process, creating gaps in their design. Scope limitations emerge in how models cater for different categories of stakeholders, disciplines, universities, and geographical contexts. Much emphasis is directed towards faculty-led research and PhD students. Although some models include master’s students, undergraduate students are largely excluded (e.g., Group Mentorship Model) ( Manabe et al., 2019). Only the Workshop-Based Model focuses on “a select few advanced undergraduate students” ( Perier et al., 2022, p. 3), yet this selective focus is itself exclusionary. The limited inclusion of certain student categories undermines sustainability and the long-term research capacity of universities. Most models concentrate on public universities, especially Makerere University, which accounts for 33.3% of all models. Overall, 67% focus on public universities, 33% are generic, and private universities are largely excluded. Only Kampala International University is included as a partner under the Group Mentorship Model ( Manabe et al., 2019). Disciplinary scope is also narrow, with 88.8% of models focusing on health research, while only one—the Busitema University Research Model—has a general scope ( Muhumuza et al., 2022). Furthermore, only three models (33%) focus specifically on Uganda, while six (67%) adopt broader contexts such as Sub-Saharan Africa or Africa as a whole.

### Limited sustainability

Although some models, such as SORT IT ( Zachariah et al., 2022) and the Workshop-Based Model ( Perier et al., 2022), improve research skills, they lack long-term embedding in university structures. Many initiatives are short-term or grant-funded, with no clear sustainability mechanisms, leading to low retention of trained staff and discontinuity in mentorship beyond the grant period.

### Resource constraints

Infrastructure and resource limitations hinder effectiveness, especially in private universities that lack strong libraries, reliable internet, and adequate funding. While all models emphasise resource input, most rely heavily on international grants, with little guidance on how universities can generate internal funds. For example, the Workshop-Based Model identified funding as its main challenge ( Perier et al., 2022). The Train-the-Trainer Model also depends largely on foreign funding, with limited local capacity and supplies ( Bebell et al., 2022). Reliance on external funding risks distorting priorities, undermining institutional ownership, and reducing local relevance. Human resources are also scarce, with few senior researchers available to mentor junior staff and students. Heavy workloads further limit their ability to engage in mentorship.

### Limited integration of SRL constructs

Strategic research leadership is only partially integrated. Six models explicitly include three SRL constructs, two models integrate four, and one integrates five. None incorporates all constructs. Vision and culture appear only once each, policy twice, while mentorship, collaboration, and resources appear three times each. This limited integration reduces effectiveness, restricting models to short-term skill enhancement rather than long-term strategic change.

### Limited research application mechanisms

Many models fail to bridge the gap between research and its application in policy or practice. Knowledge translation mechanisms are weak, meaning research often does not address immediate societal needs. For example, the Workshop-Based Model ( Perier et al., 2022) produces quality research but struggles to meet policymakers’ needs. Similarly, the Group Mentorship Model and Busitema University Research Model face challenges in linking academic output to societal impact.

### Individual vs institutional capacity gaps

Several models prioritise individual capacity building over institutional development. While they produce skilled researchers, they neglect institutional systems such as research offices and grant management structures. This disconnect renders research efforts isolated and unsustainable. The Busitema University Research Model, however, balances individual and institutional capacity by building research agendas and policies alongside individual staff development ( Muhumuza et al., 2022).

### Monitoring and evaluation gaps

Weak monitoring and evaluation frameworks limit long-term impact assessment. Models such as the Workshop-Based Model ( Perier et al., 2022) lack rigorous mechanisms, focusing instead on short-term outputs. Most rely on output-based metrics rather than tracking professional trajectories or policy impacts.

In summary, the key gaps—often highlighted in emerging frameworks such as TIMER-2C (Time, Infrastructure, Money, Expertise, Recognition, Collaboration, Culture)—show that for research capacity to improve in Ugandan universities, models must go beyond training to address systemic and institutional challenges.

## Discussion

This study systematically reviewed existing models and frameworks for research capacity enhancement in Ugandan universities, focusing on their scope, how they conceptualise strategic research leadership (SRL), the mechanisms through which they link leadership practice to measurable outcomes, and the gaps or limitations in their application. Overall, the review found that there are limited empirical models and frameworks tailored to the Ugandan university context, which explains why only nine were included. Other frameworks were either foreign-borrowed or did not meet the strict inclusion criteria.

The findings revealed that the reviewed frameworks are heavily concentrated in public universities and science-based disciplines, particularly health. This concentration means that private universities and the humanities are largely neglected. For instance, most models lean towards Makerere University, a public institution, which may explain its position as the most productive university in Uganda, consistently ranked as the best in the country and East Africa. On average, public universities are more research-productive than private ones, but with adequate and strategic support, private universities could also achieve similar levels of productivity. Additionally, major disciplines such as education remain overlooked, despite evidence that they have low research capacity in Ugandan universities ( Nakijoba & Awobamise, 2022). Students are also underrepresented, with much of the emphasis placed on faculty-led research. Most importantly, SRL is not comprehensively integrated into these frameworks. While mentorship, collaboration, and resource allocation are effectively utilised to drive outcomes such as publications, other critical SRL dimensions—such as research culture, vision, and policy—are largely overlooked. None of the frameworks integrates all six SRL dimensions. This limited integration may be a key factor restricting the effectiveness of these models at the individual level. Furthermore, sustainability remains a challenge, as many initiatives rely heavily on external donor funding and fail to adequately support student-led research, particularly at undergraduate and master’s levels. Much of the effort directed towards students is concentrated on PhD candidates.

Regarding scope, the thematic areas identified include university category (private, public, or general), target group (students, faculty, or general), region (Uganda or international), and discipline. Here, undergraduates, master’s students, private universities, and the humanities were underrepresented. On conceptualisation of SRL, all six dimensions proposed in this study—vision, collaboration and networking, mentorship, resource mobilisation and allocation, policy, and culture—emerged as thematic areas. However, integration of vision, policy, and culture was limited. The frameworks link leadership practices to measurable outcomes through structured, participatory, and context-tailored mechanisms such as structured mentorship, strategic evaluation and quality management, resource management, regional and international collaborations, and action-oriented research training. Finally, the review identified several limitations, including limited inclusion and scope, limited sustainability, resource constraints, limited integration of SRL constructs, weak research application mechanisms, individual versus institutional capacity gaps, and monitoring and evaluation weaknesses.

These findings resonate with previous studies conducted both in Uganda and internationally. For instance, strategic leadership ( Davies & Davies, 2004) and research leadership ( Flinders, 2022) have been identified as key drivers of institutional research and related interventions. Leadership is also widely recognised as a central variable for strengthening knowledge management, research, and organisational performance ( Huenneke et al., 2017; Mardian et al., 2025; Nyamboga, 2026; Wibowo et al., 2025). However, strategic research leadership itself has not been extensively explored.

International collaboration and partnerships are also cited as important factors for enhancing research capacity, particularly among postdoctoral researchers ( Amegee et al., 2023). Collaboration similarly emerged as a key theme in the reviewed models and frameworks. Other components such as resources, infrastructure, mentorship, and training are consistently supported by earlier studies ( Cloete et al., 2015; Hiire et al., 2020; Huenneke et al., 2017; Trotter et al., 2014; Obuku et al., 2021). For example, Obuku et al. (2021) highlight insufficient mentorship and supervision as major challenges faced by postgraduate students, while Kiweewa et al. (2024) argue that limited research grants significantly constrain research capacity and productivity.

The findings not only highlight gaps specific to Uganda but also mirror global trends in research capacity building. For instance, this study found that 88.8% of the reviewed models and frameworks focus on health-related research, often neglecting the humanities. This reflects broader literature from the World Bank (2023) and Rouleau (2024), which notes a global tilt towards health and STEM interventions driven by funding agendas. The dominance of flagship public universities such as Makerere University in enhancing research capacity and productivity—creating equity gaps with private universities trailing behind—is also evident in studies across Sub-Saharan Africa, Latin America, and Asia ( Matumba et al., 2026; Whyte & Whyte, 2015).

Some scholars, however, support the concentration of research capacity building and resources in flagship public universities and health sciences, arguing that this is a strategic necessity in low-resource nations. For example, Altbach (2013) and Shin (2013) suggest that developing countries should concentrate scarce resources on a few world-class public institutions to achieve global competitiveness, rather than dispersing funds across private universities. Similarly, Cloete et al. (2018) contend that prioritising STEM and health fields directly addresses urgent national developmental goals, thereby justifying the exclusion of the humanities.

Finally, this study extends the warnings of Samoff (2007) regarding donor dependency, arguing that it undermines sustainability, independence, and the development of context-specific interventions.

Regardless, we recognise that this review has its own limitations relating to exclusion criteria, methodology, and scope. Firstly, the availability, quality, and scope of existing studies posed major challenges. As a result, only nine studies were reviewed. The inclusion period was restricted to between 1 January 2015 and 8 April 2026, which may have led to the exclusion of some relevant studies. This timeframe was chosen to ensure that the models and frameworks considered aligned with current or very recent trends and knowledge. Furthermore, the study excluded grey literature, focusing solely on empirical peer-reviewed records. This may have overlooked innovative practices documented outside journals. Such publication bias favours successful models and frameworks over failed ones. In addition, the qualitative design emphasises thematic synthesis rather than measurable impact, as would be possible in meta-analyses. Reliance on global databases such as Google Scholar, Semantic Scholar, and ProQuest also risks missing smaller Ugandan studies. The study represents a snapshot in time, meaning that some of the reviewed models and frameworks may already be evolving. Moreover, the review focused on formal models and frameworks, which may have excluded more organic and informal leadership practices occurring within universities but not yet formalised into named models. Finally, the interpretive coding of SRL dimensions may have introduced an element of subjectivity.

Most importantly, the study has implications for theory, policy, practice, and future research, highlighting the need for systemic change in how research capacity enhancement is fostered in Uganda and beyond, particularly in low-income contexts. Practically, the findings provide actionable insights for university leaders and policymakers seeking to reform research ecosystems in resource-constrained settings. For researchers, educational policymakers, and university research leaders, the results suggest that models and frameworks should more comprehensively integrate SRL practices. Since leadership is the engine of any organisation, and strategic leadership has been recommended as an effective dimension for organisational success, strategic research leadership can ensure the effectiveness of research capacity enhancement interventions in Ugandan universities.

There is also a need to focus on major disciplines such as education, which have some of the highest enrolments. Developing models and frameworks that target these disciplines is likely to be more impactful and have a high multiplier effect. Consequently, this systematic review lays the foundation for empirically developing and validating a context-specific SRL model to strengthen educational research capacity among students in Ugandan private universities, as these remain the most neglected categories in existing models and frameworks.

Furthermore, government policymakers such as the Ministry of Education and Sports, the National Council for Higher Education, the Uganda National Council for Science and Technology, and the Ministry of Science, Technology and Innovation should comprehensively integrate private universities and students into policies that support research. This may include deliberate, proactive, intentional, and long-term interventions targeting research vision development, research policy development, mentorship, resource allocation, collaborations, and building robust and sustainable research cultures. Private universities can make significant contributions to research, innovation, and sustainable development if they receive the same support as public universities. At the policy level, the study highlights the necessity for government and educational bodies to address the research equity gap by extending support to private universities, students, and humanities disciplines in the form of funding.

There is also a need to reduce over-reliance on donor funding by increasing national investment in research and development (R&D) to at least the recommended 1% of GDP. Currently, Uganda invests approximately 0.31% to 0.44% of its GDP in R&D. Increasing this investment would benefit private universities, reduce dependence on foreign funding, and create sustainable internal research frameworks that address national interests.

In terms of advancing theory, this study contributes to intellectual discourse on research leadership and capacity building by examining models and frameworks in Ugandan universities and proposing SRL as a key factor for creating sustainable and strong research capacities. SRL is thus a crucial leadership dimension that can effectively address research capacity gaps in Ugandan universities. The integration of strategic leadership and research leadership to form a new, under-researched concept—SRL—is another significant contribution of this study. It enhances understanding of research capacity challenges in universities by foregrounding SRL, student-led research, research equity, and inclusion. The study also recognises and elevates the strategic contribution of students to research productivity, equity, and impact in universities, thereby advancing inclusion and equity as factors that can create more robust research cultures in higher education institutions.

Finally, the study sets a new agenda for future research, calling for empirical, context-specific, and inclusive frameworks that cater for neglected student levels, disciplines, and university categories. For instance, there is a need to develop student-centred frameworks that include undergraduate and master’s levels and expand focus to underserved humanities and social sciences. Other areas requiring further empirical examination include fostering research equity and inclusiveness, and exploring the relationship between SRL constructs and research capacity enhancement in Ugandan universities, as these could provide strong foundations for building more effective frameworks and models.

## Conclusion

There is an urgent need for a more inclusive and context-specific framework that fully integrates SRL to foster a sustainable and locally grounded research environment in Ugandan universities. At present, the existing models and frameworks for research capacity enhancement lack true equity and inclusivity, as they demonstrate inherent biases towards certain institutions, disciplines, and stakeholder groups. Prioritising research equity and inclusion within universities is vital because it democratises knowledge creation, unlocks untapped potential, enhances productivity, and ensures research addresses the diverse needs of society rather than serving only privileged institutions and groups. Without inclusive frameworks, systemic biases limit innovation and genuine capacity building across the broader academic landscape. Comprehensively integrating all dimensions of SRL would also improve the efficiency of research capacity enhancement in universities, as it provides a proactive and holistic approach that strengthens sustainability, resilience, agility, competitiveness, organisational culture, and forward progress. Ultimately, research capacity enhancement transforms universities from fragmented teaching institutions into cohesive and competitive research hubs. In the long run, it fosters innovation and sustainable development. Consequently, this study holds relevance for universities seeking to reform research cultures through leadership innovation, both in Uganda and in similar contexts across Sub-Saharan Africa and the wider developing world, where serious challenges undermine research productivity and impact. The findings can inform actionable policies to address underdeveloped research capacities in universities and guide future research aimed at developing more robust and effective models and frameworks for research capacity enhancement.

## Data Availability

The project contains the following underlying data: https://doi.org/10.6084/m9.figshare.32418141 ( Kule, 2026a). Data are available under the terms of the Creative Commons Zero “No rights reserved” data waiver (CC0). Figshare: Kule, J. (2026b). PRISMA 2020 flowchart for the study titled strategic research leadership and research capacity enhancement in Ugandan universities: a systematic review. https://doi.org/10.6084/m9.figshare.32414832 Figshare: Kule, J. (2026c). PRISMA 2020 checklist for: Strategic research leadership and research capacity enhancement in Ugandan universities: a systematic review. https://doi.org/10.6084/m9.figshare.32353485 Figshare: Kule, J. (2026d). PRISMA 2020 Abstract checklist for: Strategic research leadership and research capacity enhancement in Ugandan universities: a systematic review. https://doi.org/10.6084/m9.figshare.32353566
