# Supplementary material for: Strategic research leadership and research capacity enhancement in Ugandan universities: a systematic review
Source: F1000Res. 2026 Aug 11;15:902. Originally published 2026 Jun 9. [Version 2] doi: 10.12688/f1000research.183310.2 (PMC13638080; doi:10.12688/f1000research.183310.2)
Supplement: Peer review history

# Reviewer report: Reviewer response for version 2

**DOI:** 10.5256/f1000research.207936.r492867

**Published:** 2026-08-17

**Authors:** Nadia Tagoe

**Affiliations:** 1 Kwame Nkrumah University of Science and Technology, Kumasi, Ghana

This systematic review examines how research capacity enhancement models and frameworks in Ugandan universities foster a sustainable and locally grounded research environment. The review addresses a topical issue, given the persistent gaps in research capacity in Africa. The review emphasizes strategic research leadership as a critical dimension of research capacity and its role in enhancing capacity, particularly within university contexts. The study has clearly defined research questions which it adequately addresses. The authors premise their review on a holistic consideration of research capacity and strategic research leadership, hence highlighting capacity elements that include vision, culture, sustainability and disciplinary and stakeholder coverage.  The paper is also intentional about its context-specific focus, on Uganda and universities, to provide contextually grounded findings to enhance applicability over universally applied solutions. The review provides a foundation for empirically developing a research enhancement framework that centres strategic research leadership and addresses the identified gaps. While the revised version of the paper is strengthened, I have a few comments for the authors to consider.

 The authors should provide a clearer justification for the focus on research leadership, including its significance within research capacity strengthening.  While strategic research leadership (SRL) is described later, it would be helpful to first establish the conceptual basis and importance of research leadership (RL) before integrating it with strategic leadership (SL) to introduce SRL.

 It is important to precede the introduction of strategic leadership (SL) with a clearer articulation of the gaps in existing research leadership (RL) approaches that SRL seeks to address, and explain how SRL advances current thinking and practice. This would demonstrate more clearly that the proposed innovation builds on, rather than departs from, the existing evidence base.

 Clarify whether SRL considered at the individual, institutional, or both levels. Some dimensions, such as mentoring and collaboration, appear more individually oriented, while others are institutional. It would be important to establish this multilevel conceptualisation from the outset and explain how it informed the extraction and analysis of data. Greater conceptual clarity around SRL would be valuable in clearly delineating its key elements and identifying which of these are not adequately addressed within existing frameworks.

 In discussing the SRL constructs, it is important to highlight the emphasis on more tangible and research process-related capacities compared to less tangible dimensions such as vision and research culture.

 It would also be useful to explore whether the frameworks were project-based or institutionally driven, and whether they reflected short- or longer-term approaches as this would highlight institutional embeddedness and sustainability issues.

 The discussion could also be strengthened by more explicitly situating the findings within the Ugandan research capacity context.

Are the rationale for, and objectives of, the Systematic Review clearly stated?

Yes

Is the statistical analysis and its interpretation appropriate?

Not applicable

If this is a Living Systematic Review, is the ‘living’ method appropriate and is the search schedule clearly defined and justified? (‘Living Systematic Review’ or a variation of this term should be included in the title.)

Not applicable

Are sufficient details of the methods and analysis provided to allow replication by others?

Yes

Are the conclusions drawn adequately supported by the results presented in the review?

Yes

Reviewer Expertise:

Research capacity strengthening, research systems, ethics and justice

I confirm that I have read this submission and believe that I have an appropriate level of expertise to confirm that it is of an acceptable scientific standard, however I have significant reservations, as outlined above.

# Reviewer report: Reviewer response for version 2

**DOI:** 10.5256/f1000research.207936.r509939

**Published:** 2026-08-12

**Authors:** Justin Pulford

**Affiliations:** 1 Liverpool School of Tropical Medicine, Liverpool, UK

I have no further comments to make

Are the rationale for, and objectives of, the Systematic Review clearly stated?

Yes

Is the statistical analysis and its interpretation appropriate?

Not applicable

If this is a Living Systematic Review, is the ‘living’ method appropriate and is the search schedule clearly defined and justified? (‘Living Systematic Review’ or a variation of this term should be included in the title.)

Not applicable

Are sufficient details of the methods and analysis provided to allow replication by others?

Yes

Are the conclusions drawn adequately supported by the results presented in the review?

Partly

Reviewer Expertise:

Research system strengthening

I confirm that I have read this submission and believe that I have an appropriate level of expertise to confirm that it is of an acceptable scientific standard.

# Reviewer report: Reviewer response for version 1

**DOI:** 10.5256/f1000research.202348.r492868

**Published:** 2026-07-21

**Authors:** Sandy Oliver

**Affiliations:** 1 University College London, London, UK

This study addresses an important question: namely what research capacity enhancement models or frameworks fully integrate strategic research leadership to foster a sustainable and locally grounded research environment in Ugandan universities? This overarching question is my interpretation of the work, based on the authors’ listing of four research questions and their conclusion. I would prefer a clearer indication of the overarching research question.

 This is important for Uganda and other low- and middle-income countries (LMICs) because of the of the ‘disconnect... between broad global capacity-building models and the highly specific, resource-constrained realities faced by universities in low- and middle-income countries (LMICs)’. The authors may like to strengthen their argument for the global importance of this work by noting that Uganda is one of the countries most vulnerable to climate change, and therefore at the forefront of climate change and offering opportunities for research addressing the greatest challenge to the whole world. They can also strengthen their argument for adapting Strategic Research Leadership strategies for private universities and the humanities by noting how their larger student numbers would help citizen science in the area of mitigating or adapting to climate change.

 This study has two important design strengths. First is its combination of two concepts: strategic leadership and research leadership, thereby ‘linking leadership practice to measurable research capacity outcomes’. Second is its application of systematic review methods using PRISMA 2020 guidelines. The inclusion criterion of ‘Focusing on Ugandan universities’ makes the work narrower and manageable, with a clear and suitable scope for this innovative thinking.

 The authors found that most research capacity enhancement models and frameworks were directed towards public universities (particularly health), with private universities, students, and the humanities being underrepresented. Yet Uganda and other LMICs rely heavily on private universities, to accommodate the rapid expansion of secondary education graduates. With both authors members of a humanities faculty (education), one at a private university and another at a public university, they would be well placed to critique the frameworks in terms of how well they might suit the humanities disciplines and private universities, how the frameworks might be adapted to better suit the humanities and research that is led by or conducted by students, and what might be gained from embracing the humanities and universities that emphasise student activities.

Are the rationale for, and objectives of, the Systematic Review clearly stated?

Yes

Is the statistical analysis and its interpretation appropriate?

Not applicable

If this is a Living Systematic Review, is the ‘living’ method appropriate and is the search schedule clearly defined and justified? (‘Living Systematic Review’ or a variation of this term should be included in the title.)

Not applicable

Are sufficient details of the methods and analysis provided to allow replication by others?

Yes

Are the conclusions drawn adequately supported by the results presented in the review?

Yes

Reviewer Expertise:

The interface between academia and the wider world

I confirm that I have read this submission and believe that I have an appropriate level of expertise to confirm that it is of an acceptable scientific standard, however I have significant reservations, as outlined above.

# Response

**Published:** 2026-08-04

**Authors:** Jerald Kule

**Affiliations:** Education, Ibanda University, Ibanda, Uganda, Uganda

We sincerely thank the reviewer for thoughtful and constructive comments on study. We appreciate both the positive assessment and the valuable suggestions provided. The comments have helped us to improve the clarity, interpretation and overall quality of our article. We have carefully considered each point raised, and revised the article accordingly. Below we provide responses to the specific comments:

 Comment 1: …a clear indication of the overarching research question.

 We thank the reviewer for this helpful observation. We agree that the overarching research question was not stated explicitly enough in the previous version. Therefore, we have revised the article to clearly indicate this question in the abstract, introduction, methodology and discussion sections. However, we have also retained the four specific review questions that guided the analysis. We believe this has improved the clarity and coherence of the review.

 Comment 2: …strengthening our argument for the global importance of the work…and adapting SRL practices for private universities and the humanities

 We appreciate this valuable suggestion. We have strengthened the discussion to highlight the wider relevance of the study, especially its contribution to addressing global challenges such as climate change and quality education. The discussion also now reflects the role of private universities, the humanities, and student-led research, and how future RC enhancement models and frameworks can be better adapted to these contexts. The revised discussion also highlights their potential contribution to locally relevant and globally significant research.

 Comment 3: …the two important design strengths… its combination of two concepts: strategic leadership and research leadership, ‘linking leadership practice to measurable research capacity outcomes.’ And ... application of systematic review methods using PRISMA 2020 guidelines. The inclusion criterion of ‘Focusing on Ugandan universities’ makes the work narrower and manageable, with a clear and suitable scope for this innovative thinking.

 We sincerely thank the reviewer for these encouraging remarks. We are pleased that the integration of strategic leadership and research leadership to link leadership practice to measurable research capacity outcomes, the use of the PRISMA 2020 guidelines, and the Uganda-specific focus of the review were recognised as strengths of the study. We appreciate this positive assessment.

 Comment 4: …critique the frameworks in terms of how well they might suit the humanities disciplines and private universities, how the frameworks might be adapted to better suit the humanities and research that is led by or conducted by students, and what might be gained from embracing the humanities and universities that emphasise student activities

 We thank the reviewer for this insightful comment. We have expanded the discussion section to provide a more critical reflection on the suitability of existing research capacity enhancement models and frameworks for private universities, the humanities, and student-led research. We also discuss why direct transfer of models developed in research-intensive public universities may not always be appropriate. We have also highlighted the need for future models and frameworks to place greater emphasis on contextual adaptation, student-led research, interdisciplinary collaboration, community engagement, and the realities of private universities.

# Reviewer report: Reviewer response for version 1

**DOI:** 10.5256/f1000research.202348.r495523

**Published:** 2026-07-20

**Authors:** Justin Pulford

**Affiliations:** 1 Liverpool School of Tropical Medicine, Liverpool, UK

This is a well written manuscript exploring a neglected, yet important topic; namely, the role and contribution of strategic research leadership within university-focused research capacity strengthening frameworks and models.  The authors employed a systematic review methodology to undertake this exploration and focused their efforts specifically within a Ugandan context.  It is encouraging to read that the review findings are intended to “lay the foundation for empirically developing and validating a context-specific SRL model to strengthen educational research capacity among students in Ugandan private universities.”   I am sure many of the reported findings will assist in this endeavour and it is laudable that the authors have sought to adopt a systematic, evidence-informed approach to doing this; in my experience, many research capacity strengthening efforts are undertaken on assumptions of good practice as opposed to a firm evidence-informed basis. Having said this, I do have some questions or concerns re the review design and reported findings.  I have outlined these below for the authors consideration and possible clarification.  They are offered in the spirit of strengthening both the manuscript and any subsequent “context-specific SRL model” that may follow.

 Strategic research leadership (SRL)

 Your review has identified six dimensions of SRL as conceptualised across the 9 frameworks/models.  However, these six dimensions may not be exhaustive nor may they even be the most important; they are simply those articulated within, or derived from, the models identified.  I would strongly recommend exploring established frameworks/models specific to SRL (as opposed to research capacity strengthening), which may then even be used to assess the identified capacity strengthening models against (e.g. to what extent do the SRL dimensions identified in your review map onto SRL dimensions reported elsewhere).  In short, I am not convinced this review is the best approach to identifying important SRL dimensions; rather, it simply identifies those that have been reported within a research capacity strengthening model.

 Research capacity strengthening models/frameworks

 Your review specifically set out to identify research capacity strengthening (RCS) models/frameworks applied in a Ugandan university context; however, I am not convinced the 9 studies identified are presenting RCS models/frameworks as opposed to RCS initiatives/interventions.  Ultimately, I guess this depends on how you define a ‘model’ or ‘framework’ and it would be helpful if you did so here.  I would understand a model/framework as a holistic conceptualisation as to how to systematically strengthen the research capacity of a university.  Many of the papers you identified are simply describing an approach to strengthening a specific-type of research capacity (e.g. SORT IT primarily aims to strengthen implementation research capacity of healthcare workers). I would consider these ‘RCS interventions’ (as opposed to RCS models/frameworks) that are intentionally limited in scope – they were never designed to systematically strengthen the full range of required research capacities at a University level, yet you critique them on this basis (Q4 in ‘results’). 

 Related to this, there are a several RCS conceptual models/frameworks that do attempt to map out the full range of research capacities that may be needed within a university context; however, these are very rarely country specific and would have been excluded on the basis of your review methodology.  I’m not convinced the research capacities a university in Uganda needs would differ greatly from the research capacities universities in other African countries (or, indeed, globally) would need and, as such, I do not understand why you excluded papers on this basis. 

 Alternatively, if you were committed to understanding RCS models/frameworks unique to Uganda, then limiting your search to peer reviewed papers only is problematic.  This would have excluded any government initiatives designed to strengthen national research capacity as well as many other RCS initiatives (whether supported nationally or externally) that are not described in the published literature (and most will not be).

 Defining ‘research capacity enhancement’

 Your paper favours the term ‘research capacity enhancement’ over other related terms such as ‘research capacity strengthening’, ‘research capacity building’ or ‘research capacity development’.  Is there a specific reason for this? I.e. do you understand research capacity enhancement to mean something different to the other related terms or do you use them interchangeably? More importantly, how do you define ‘research capacity enhancement’ and what does that mean at the university-level?  I.e. what research capacities does your definition pertain to?  It would be helpful if you were able to articulate what you consider a well capacitated university to look like as without this definition/clarity, it is not always clear as to what it is you are trying to achieve.  In some instances, you refer to “educational research capacity among students”, in others you refer to faculty and in others you distinguish between individual and institutional level capacities.  It may be that your understanding and aims pertain to all of these things, but without offering a clear definition then it is difficult for the reader to follow.

 Minor points

 At sections in the introduction, you draw a distinction between ‘Africa’ and ‘SSA’.  I’m not sure this is needed or helpful, especially as your paper focuses solely on Uganda anyway.  I suggest you do away with this distinction and just use either Africa or SSA as the wider contextual background to your review.

 Also in the introduction you present SRL as a proposed solution to inadequate research capacity.  You may need to moderate this claim a little, SRL may be essential to strengthening research capacity, but will be insufficient on its own.

Are the rationale for, and objectives of, the Systematic Review clearly stated?

Yes

Is the statistical analysis and its interpretation appropriate?

Not applicable

If this is a Living Systematic Review, is the ‘living’ method appropriate and is the search schedule clearly defined and justified? (‘Living Systematic Review’ or a variation of this term should be included in the title.)

Not applicable

Are sufficient details of the methods and analysis provided to allow replication by others?

Yes

Are the conclusions drawn adequately supported by the results presented in the review?

Partly

Reviewer Expertise:

Research system strengthening

I confirm that I have read this submission and believe that I have an appropriate level of expertise to confirm that it is of an acceptable scientific standard, however I have significant reservations, as outlined above.

# Response

**Published:** 2026-08-04

**Authors:** Jerald Kule

**Affiliations:** Education, Ibanda University, Ibanda, Uganda, Uganda

We would like to sincerely thank the reviewer for the thoughtful, constructive, and encouraging comments on our article. We greatly appreciate the positive assessment of the study and the insightful suggestions for improving both the review and the future development of a context-specific SRL model. We have carefully considered each comment and revised the manuscript accordingly. Our responses to the specific comments are provided below:

 Comment 1: Strategic research leadership (SRL)

 We thank the reviewer for this thoughtful and important observation. We would like to clarify that the six SRL dimensions were not derived inductively from the reviewed research capacity enhancement models and frameworks. Rather, they were conceptualised from established strategic leadership (Davis & Davis 2004, 2005, 2006, 2010; Samimi et al., 2022) and research leadership literature (Flinders, 2022) and used as the analytical lens for examining how the reviewed models and frameworks conceptualised and integrated SRL. This clarification has been added in the introduction section of the paper. However, we agree that these dimensions should not be regarded as exhaustive or definitive. We also acknowledge that further conceptual and empirical work is needed to refine and validate the conceptualisation of SRL.

 Comment 2: Research capacity strengthening models/frameworks

 We appreciate this valuable comment. In the revised second version of the article, we have clarified the operational definition of research capacity enhancement models and frameworks used in this review. We have also acknowledged that the included studies varied in scope, with some representing broader institutional frameworks and others focusing on specific research capacity enhancement interventions. We have also strengthened the justification for restricting the review to Ugandan universities by explaining the importance of context-specific policy, governance, funding, and institutional environments when examining research capacity enhancement. We have also acknowledge that restricting the review to peer-reviewed literature may have excluded relevant government initiatives, institutional frameworks, and other unpublished evidence. All these acknowledgements have been captured in the expanded limitations section of the revised version.

 Comment 3: Defining ‘research capacity enhancement’

 We are thankful for this helpful suggestion. The article has been revised to provide a clear operational definition of research capacity enhancement and explained our use of this term in relation to other commonly used terms (development, strengthening, building).

 Comment 4: Minor points

 Thank you for these helpful observations. The introduction has been revised to improve clarity by removing the unnecessary distinction between Africa and Sub-Saharan Africa. We have maintained Africa as the wider contextual background. We have also moderated the claim about SRL, presenting it as an “important leadership dimension capable of enhancing research capacity”.
